# Mitogenome Diversity and Maternal Origins of Guangxi Buffalo Breeds

**DOI:** 10.3390/ani10040547

**Published:** 2020-03-25

**Authors:** Ting Sun, Guangyun Huang, Junli Sun, Zihao Wang, Shaohua Teng, Yanhong Cao, Quratulain Hanif, Ningbo Chen, Chuzhao Lei, Yuying Liao

**Affiliations:** 1 Animal Husbandry Institute of Guangxi Zhuang Autonomous Region, Guangxi Key Laboratory of Livestock Genetic Improvement, Nanning 530001, China; sunting_sim07@163.com (T.S.); hgy699@163.com (G.H.); sjn313@126.com (J.S.); wangzh761006@163.com (Z.W.); tsh088@126.com (S.T.); caoyh610@163.com (Y.C.); 2 College of Animal Science and Technology, Northwest A&F University, Yangling 712100, China; ningboch@126.com (N.C.); leichuzhao1118@nwafu.edu.cn (C.L.); 3 National Institute for Biotechnology and Genetic Engineering, Pakistan Institute of Engineering and Applied Sciences, Faisalabad 577, Pakistan; micro32uvas@gmail.com

**Keywords:** buffalo, mtDNA, genetic diversity, maternal origin

## Abstract

**Simple Summary:**

Mitochondrial DNA (mtDNA) analysis is an important tool to assess the maternal origin and phylogeny of domestic animals. Fuzhong and Xilin buffalo are two native buffalo breeds in Guangxi, a province in the southwest of China that belongs to the major hotspots of swamp buffalo domestication centers. In this study, we sequenced the complete mitochondrial genomes of two buffalo breeds in Guangxi Province to perform the phylogenetic analysis.

**Abstract:**

Guangxi Province, in the southwest of China, is one of the putative migratory corridors or domestication centers for swamp buffalo. In this study, we investigated the evolutionary status of two Guangxi native buffalo breeds (Fuzhong buffalo, *n* = 15; Xilin buffalo, *n* = 25) based on the complete mitogenome sequencing. Our results revealed rich genetic diversity in the two buffalo breeds. We detected five haplogroups (SA1, SA2, SB1, SB2, SB3) in the two Guangxi buffalo breeds, and the haplogroup SB3 in the Fuzhong buffalo. Our results showed that the haplogroup SA1 was associated with the major domestication event that involved population expansion in Guangxi buffalo. In conclusion, our findings revealed a high level of maternal genetic diversity and the phylogenetic pattern of the two Guangxi buffalo breeds.

## 1. Introduction

The domestic Asian water buffalo (*Bubalus bubalis*)*,* a valuable species in tropical and subtropical climates, provides meat, milk, and hides [1]. According to the morphological, biological characteristics, and chromosome karyotype, the domestic Asian buffalo are divided into two types: river buffalo (2n = 50) and swamp buffalo (2n = 48) [2,3]. The river buffalo is mainly used as a dairy animal and is distributed from Western India to the Mediterranean areas. The swamp buffalo is traditionally raised as a draught animal for rice cultivation, and is mainly bred in extensive rural areas in Northeast India, Southeast Asia and South China. Previous studies have reported that the two types of buffalo descended from different wild Asian water buffalo in separate geographical regions [4,5]. The river buffalo was domesticated in the western region of the Indian subcontinent, and then spread to the west. The swamp buffalo was domesticated in the China/Indochina border region and later spread to the other regions [4,6].

Mitochondrial DNA (mtDNA) is a very useful tool to investigate genetic diversity, phylogeny, and maternal origins of domesticated animals. However, to date, most studies have been limited to the mitochondrial D-loop region and *cytochrome b* gene [4,6,7,8,9]. Previous studies of mtDNA showed that swamp buffalo can be assigned into the five previously defined haplogroups: Two major haplogroups (SA and SB with various subclades) and three rare ones (SC, SD, and SE) [4,10]. Wang et al. defined the mtDNA subhaplogroups of swamp buffalo using the 16 kb mitogenome sequences as follows: SA1 (SA1a, SA1a1, SA1a2, and SA1a3); SB1 (SB1a, SB1a1, SB1a2, and SB1b); SB2 (SB2a and SB2b); SB3 (SB3a and SB3a1); and SD (SD1 and SD2) [10]. Guangxi is located in the southwest of China, which belongs to the domestication area of swamp buffalo. In Guangxi, there are two prominent native buffalo breeds: Fuzhong and Xilin buffalo. The Fuzhong buffalo is mainly distributed in the west of Guangxi Province, while the Xilin buffalo is in the east of Guangxi Province. However, there are limited studies about the two breeds. The purpose of the present study was to investigate the maternal origins and genetic diversity of the two Guangxi buffalo breeds by analysis of the complete mitochondrial sequences.

## 2. Materials and Methods

### 2.1. Sample Collection and Sequencing

We sampled a total of 40 swamp buffalo from 2 buffalo breeds in Guangxi Province, including 15 Fuzhong buffalo and 25 Xilin buffalo. All experimental procedures were performed in accordance with the Regulations for the Administration of Affairs Concerning Experimental Animals approved by the State Council of the People’s Republic of China. This study was approved by Institutional Animal Care and Use Committee of Northwest A&F University (permit number: NWAFAC1019). Genomic DNA was extracted from ear tissue using the standard phenol-chloroform protocol. Sequencing was performed on an Illumina HiSeq 2000 at the Novogene Bioinformatics Technology Co., Ltd., Beijing, China. For each individual, 1–15 μg of DNA was used to construct libraries using the NEBNext^®^ Ultra^TM^ (Illumina, San Diego, CA, USA), according to the manufacturer’s recommendations. Firstly, each DNA sample was fragmented to a size of 350 bp, and then end-polished, A-tailed and ligated with the full-length adaptor to perform sequencing with further PCR amplification. Secondly, the PCR products were purified by an AMPure XP system (Beckman Coulter, Beverly, MA, USA), the size distribution of libraries was analyzed using the Agilent 2100 Bioanalyzer (Agilent Technologies, Palo Alto, CA, USA), and the libraries were quantified by real-time PCR. Then, the index-coded samples were clustered by the cBotCluster Generation System (Illumina, San Diego, CA, USA), according to the manufacturer’s instructions. Lastly, libraries were sequenced on an Illumina HiSeq platform.

### 2.2. Reconstruction of Mitochondrial Genomes

To assemble the complete mitochondrial genomes (mtDNA), we mapped the sequencing reads to the swamp buffalo mitochondrial genome (NC_006295.1) using BWA-MEM (v0.7.13-r1126) with the default settings [11]. Since the mitochondrial genomes are circular, we added 30 bp of the first base pairs to the end of the reference to ensure equal coverage of the sequences across the mtDNA. The BAM alignments were transformed into fastq, and then mitochondrial sequences were assembed using Mapping Iterative Assembler (MIA) V 1.0 with the parameters: -H 1 -F -i -c -r [12]. The average depth-of-coverage was 1642.45 X, ranging from 161.78 X to 2679.85 X (Table S1). The sequences have been deposited in GenBank under the accession numbers MT186704–MT186743. A total of 14 reference complete mitochondrial genomes corresponding to individuals of known haplogroup affiliation were retrieved from a previous study [10], and one from NCBI (AF547270).

### 2.3. Data Analysis

Haplotype diversity (H), nucleotide diversity (π), numbers of haplotypes, variable sites, and the average number of nucleotide differences (k) were estimated using DnaSP 5.0 [13]. The neighbor-joining (NJ) tree was constructed using MEGA 6.0 [14], and the reliability of the tree topology was assessed by 1000 bootstrap replications. The maximum likelihood (ML) tree was constructed by IQ-tree-1.6.6.a [15] using the TIM2 + F + R3 model and 1000 bootstrap replicates under the following parameters: -s sequences.phy -m TIM2 + F + R3 -nt 4 -bb 1000 -redo. Nucleotide substitution and site heterogeneity models were also estimated using IQ-tree-1.6.6.a [15] with the following parameters: -s sequences.phy -m MF -nt 4. Lastly, the network of the complete mitochondrial genomes was constructed using pegas [16].

## 3. Results

### 3.1. MtDNA Sequence Variation and Genetic Diversity

**Figure 1:**
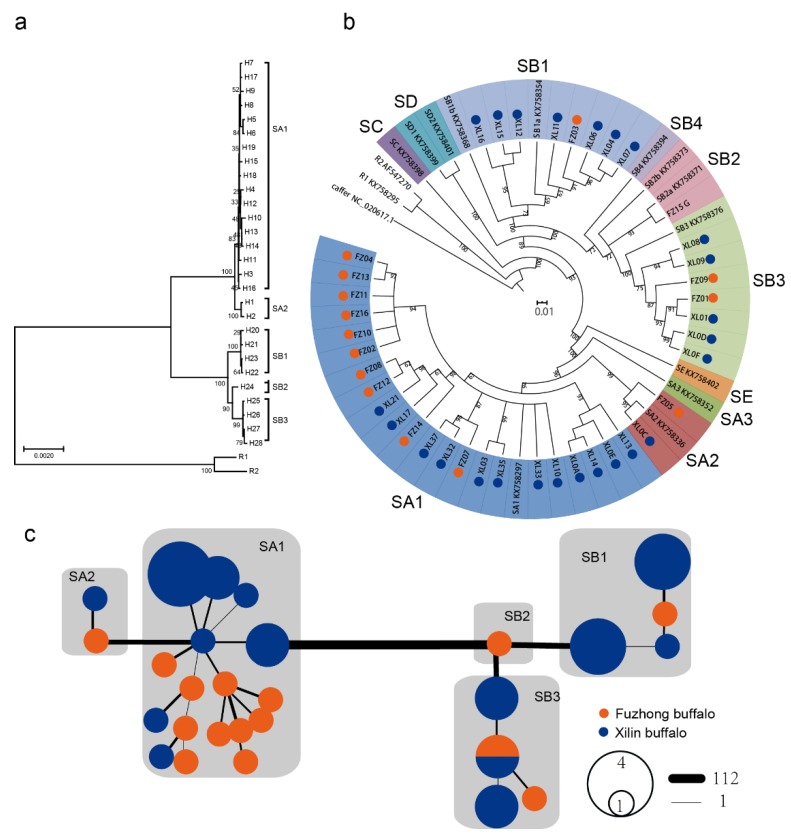
(**a**) A neighbor-joining (NJ) tree of 28 haplotypes in Guangxi buffalo. The values on the branches represent the bootstrap values supported based on 1000 replications. (**b**) A maximum likelihood (ML) tree of 40 Guangxi buffalo and 15 buffalo reference sequences. The ML tree was rooted by *Syncerus caffer* (NC_020617.1), which was used as an outgroup. (**c**) Mitogenome network of 40 buffalo. The size of the circle represents the individual number of each haplotype. The width of the edges is proportional to the number of pairwise differences between the joined haplotypes.

In this study, we analyzed the sequence variation of 40 complete mitogenome sequences (16,355 to 16,359 bp) from two Guangxi buffalo breeds to assess their mtDNA genetic diversity, phylogeny, and maternal origin. There were 164 variable sites detected among the 40 samples, which defined a total of 28 haplotypes (Figure 1a, Table S2). The mtDNA polymorphic sites for the two Guangxi buffalo breeds are listed in Table S1. Among the 28 detected haplotypes, only H27 was shared by the Fuzhong and Xilin buffalo breeds. In the Fuzhong buffalo, a total of 13 specific haplotypes were detected, and all these were only observed once. In the Xilin buffalo, a total of 13 specific haplotypes were detected. Among these haplotypes for the Xilin buffalo, six haplotypes were only observed once, while the most frequent haplotypes, H11 and H20, occurred four and three times, respectively (Table S2).

Next, we estimated the mtDNA genetic diversity and haplogroup frequencies in the two Guangxi buffalo breeds (Table 1). The haplotype diversity of the Fuzhong buffalo (1.000 ± 0.024) was higher than that of the Xilin buffalo (0.947 ± 0.023), revealing the higher genetic diversity of the Fuzhong buffalo. More detailed information about genetic diversity estimates, including the number of variable sites (S), the number of haplotypes (H), and nucleotide diversity (Pi ± SE), is provided in Table 1.

### 3.2. Population Phylogenetic Analysis

We constructed the ML tree using IQ-tree based on 40 buffalo mitogenomes, 13 representative sequences of swamp mtDNA haplogroups (SA1, SA2, SA3, SB1, SB2, SB3, SB4, SC, SD, SE) and 2 riverine sequences; *Syncerus caffer* (accession no. NC_020617.1) was used as an outgroup (Figure 1b). Phylogenetic analyses showed that all the Guangxi buffalo belong to the swamp buffalo. The 40 Guangxi buffalo can be divided into two major swamp haplogroups (SA and SB with various subclades), and no rare haplogroups (SC, SD, SE) were detected (Table 1, Figure 1a–c). Haplogroup SA1 was extremely frequent (60%) in the two Guangxi buffalo breeds, while SB1 and SB3 occurred mainly in the Xilin buffalo. We also found that the haplogroup SB2 was only detected in the Fuzhong buffalo.

In order to focus on the phylogenetics among Guangxi buffalo, a network of 40 buffalo samples was constructed (Figure 1c). As expected, the network identified haplogroups SA and SB, which were separated by 112 variants. A total of 19 haplotypes representing 24 individuals belonged to haplogroup SA. The haplogroup SA1 showed a star-like phylogenetic relationship.

## 4. Discussion

Fuzhong and Xilin buffalo are the native Guangxi buffalo breeds that are distributed in the hotspots for potential swamp buffalo domestication [4,10]. To date, most published studies about their mtDNA have mainly focused on the mitochondrial D-loop region and the *cytochrome b* gene [4,6,7,8,9]. However, there are limited studies based on the complete mtDNA genome. In this study, we investigated the genetic diversity and maternal origin of the two Guangxi buffalo breeds, which can provide initial insights into the phylogenetic pattern of these breeds.

The phylogenetic analysis showed that all of the Guangxi buffaloes included in this study were swamp buffalo. Previous studies have identified seven frequent swamp buffalo haplogroups (SA1, SA2, SA3, SB1, SB2, SB3, SB4), together with three rare haplogroups (SC, SD, SE). Wang et al. further subdivided the mtDNA subhaplogroups for SA1, SB1, SB2, SB3, and SD [10]. In this study, we detected five haplogroups (SA1, SA2, SB1, SB2, SB3) in the Guangxi buffalo. Previous studies have detected four haplogroups (SA1, SA2, SB1, SB2) in the Fuzhong buffalo, while five haplogroups (SA1, SA2, SB1, SB2, SB3) were observed in the Xilin buffalo [4,6]. Here, we found that the SB3 haplogroup was also detected in the Fuzhong buffalo, though with only two individuals. Our results showed that the haplogroup SA1 dominated in Fuzhong (45%) and Xilin buffalo (55%), and thus were consistent with previous studies based on the mtDNA control region and *cytochrome b* gene [4,6]. Furthermore, the haplogroup SA1 showed a star-like phylogeny, which was in accordance with the results of previous studies [4,6,8,17]. The star-like phylogeny is typical of domestic species, suggestive of a past population expansion as described by previous studies [4,8,18]. Therefore, SA1 was the major domestication event in Guangxi buffalo, which is consistent with other swamp buffalo [4,6]. The results showed that SB1 and SB3 occurred mainly in the Xilin buffalo. Our results might be due to bias or compromised representation of the haplotypes with the limited sample size. With more samples, a clearer insight could be obtained.

## 5. Conclusions

In conclusion, our findings revealed a high level of maternal genetic diversity and the phylogenetic pattern of the two Guangxi buffalo breeds. Our results showed that SA1 was the major domestication event in Guangxi buffalo.

## Figures and Tables

**Table 1:** Structure and diversity of Fuzhong and Xilin buffalo.

Breed	N	S	H	Haplogroup					Hd ± SE	Pi ± SE	k
				SA1	SA2	SB1	SB2	SB3			
Fuzhong	15	156	15	10	1	1	1	2	1.000 ± 0.0006	0.00332 ± 0.00294	54.267
Xilin	25	153	14	12	1	7	0	5	0.947 ± 0.0005	0.00409 ± 0.00249	66.893
Total	40	164	28	22	2	8	1	7	0.978 ± 0.0001	0.00381 ± 0.00237	62.286

N: number of buffalo; S: number of variable sites; H: number of haplotypes; k: the average number of differences; Hd: haplotype diversity; Pi: nucleotide diversity; SE: standard error.

## Supplementary Materials

The following are available online at https://www.mdpi.com/2076-2615/10/4/547/s1, Table S1: Mapping results for 40 Guangxi buffalo mitochondrial genomes. Table S2: Mitochondrial sequence variation of Guangxi buffalo.
